# Exosomes neutralize synaptic-plasticity-disrupting activity of Aβ assemblies *in vivo*

**DOI:** 10.1186/1756-6606-6-47

**Published:** 2013-11-13

**Authors:** Kyongman An, Igor Klyubin, Youngkyu Kim, Jung Hoon Jung, Alexandra J Mably, Sean T O’Dowd, Timothy Lynch, Daniel Kanmert, Cynthia A Lemere, Gina M Finan, Joon Won Park, Tae-Wan Kim, Dominic M Walsh, Michael J Rowan, Joung-Hun Kim

**Affiliations:** 1 Department of Life Sciences, Pohang University of Science and Technology (POSTECH), Pohang, Gyungbuk 790-784, Korea; 2 Department of Pharmacology and Therapeutics, and Institute of Neuroscience, Biotechnology Building, Trinity College, Dublin 2, Ireland; 3 School of Interdisciplinary Bioscience and Bioengineering, Pohang University of Science and Technology (POSTECH), Pohang, Gyungbuk 790-784, Korea; 4 Laboratory for Neurodegenerative Research, Center for Neurologic Diseases, Brigham & Women’s Hospital, Harvard Institute of Medicine, 77 Avenue Louis Pasteur, Boston, MA 02115, USA; 5 Laboratory for Neurodegenerative Research, Conway Institute of Biomolecular and Biomedical Research, University College Dublin, Dublin 4, Ireland; 6 Dublin Neurological Institute at the Mater Misericordiae University Hospital, 57 Eccles Street, Dublin 7, Ireland; 7 Department of Pathology and Cell Biology, Taub Institute for Research on Alzheimer’s Disease and the Aging Brain, Columbia University, New York, NY 10032, USA

**Keywords:** Alzheimer’s disease, Aβ, Exosomes, Synaptic plasticity, PrP^C^

## Abstract

**Background:**

Exosomes, small extracellular vesicles of endosomal origin, have been suggested to be involved in both the metabolism and aggregation of Alzheimer’s disease (AD)-associated amyloid β-protein (Aβ). Despite their ubiquitous presence and the inclusion of components which can potentially interact with Aβ, the role of exosomes in regulating synaptic dysfunction induced by Aβ has not been explored.

**Results:**

We here provide *in vivo* evidence that exosomes derived from N2a cells or human cerebrospinal fluid can abrogate the synaptic-plasticity-disrupting activity of both synthetic and AD brain-derived Aβ. Mechanistically, this effect involves sequestration of synaptotoxic Aβ assemblies by exosomal surface proteins such as PrP^C^ rather than Aβ proteolysis.

**Conclusions:**

These data suggest that exosomes can counteract the inhibitory action of Aβ, which contributes to perpetual capability for synaptic plasticity.

## Background

Alzheimer’s disease (AD) is characterized by progressive cognitive decline [1,2]. Accumulating evidence has attributed this deficit in the cognitive capacity of patients and the potentially responsible failure in neural circuits to an increased amount of amyloid β-protein (Aβ), particularly soluble Aβ oligomers rather than fibrils [3]. To examine the mechanisms that underlie the synaptic dysfunction caused by Aβ oligomers, several laboratories have utilized a cellular correlate of learning and memory - long-term potentiation (LTP) - and have studied the effectiveness of different forms of soluble Aβ preparations including Aβ-derived diffusible ligands (ADDLs) and AD brain-derived Aβ [4-7]. As Aβ oligomers appear to execute their deleterious activities (*i.e.*, LTP impairment) by binding to their putative receptors such as p75 neurotrophin receptor, insulin receptor, and cellular prion protein (PrP^C^) [4,7-9], Aβ assemblies or their receptors have been targeted to develop effective therapeutic strategies [6,10,11]. Despite enormous efforts, however, the molecular identity and importance of intrinsic extracellular factors for regulating the activities of Aβ oligomers are still poorly understood.

In this study, we focused on one class of extracellular vesicles, exosomes, as a potential regulator of Aβ and its effects on synaptic plasticity *in vivo*. Exosomes are small (30 - 100 nm diameter) membranous vesicles that are secreted naturally into the extracellular space upon fusion of multivesicular bodies with the plasma membrane [12]. Although exosomes have been proposed to exert multiple physiological roles [13] and are also known to contain machinery to synthesize, degrade and induce aggregation of Aβ [14-16], whether these factors in exosomes increase or decrease the deleterious actions of Aβ is a matter of debate [15-18].

Direct assessment of the effect of exosomes on the activity of synaptotoxic Aβ has been impeded by the difficulty in controlling their levels *in vivo*. Here, we have manipulated the concentration of exosomes in the brain by infusing exosomes *intracerebroventricularly* (i.c.v.) and then examined their effect on Aβ-mediated impairment of synaptic plasticity. We find that exosomes neutralize the synaptic-plasticity disrupting activities of Aβ *in vivo*, and also show that these effects are primarily the result of the sequestration of Aβ oligomers via exosomal surface proteins such as PrP^C^. The potential relevance of our findings to AD is underscored by our observation that exosomes from human cerebrospinal fluid (CSF) prevent the impairment of LTP that is mediated by Aβ derived from AD brain extracts.

## Results

### Exosomes attenuated ADDL-mediated LTP inhibition

**Figure 1:**
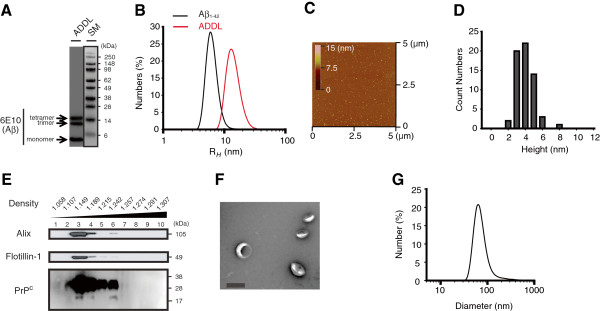
**Characterization of ADDLs and exosomes used for biochemical and physiological experiments. (A)** ADDLs were analyzed by Western blotting using 6E10. SM, size markers (kDa). **(B)** DLS particle distribution analysis of ADDLs (red line) (14.3 ± 1.1 nm) and Aβ_1-42_ freshly dissolved in 10 mM NaOH (black line) (6.4 ± 0.3 nm) was expressed as hydrodynamic radii (R_*H*_). **(C)** A tapping AFM mode image of ADDLs (X-Y, 5 x 5 μm with an *inset* displaying a z-range in color from 0 to 15 nm). (**D**) By AFM, only small (3 - 6 nm) globular structures were detected. **(E)** Exosomes isolated from the conditioned medium of N2a cells had their density 1.13 g/ml to 1.19 g/ml, and contained the exosomal marker proteins Alix, Flotilin-1 and PrP^C^. Multiple (non-, mono- or di-) glycosylated PrP^C^ proteins were detected between 20 ~ 35 kDa on SDS-PAGE. **(F)** By EM, exosomes appeared as closed vesicles of 30-120 nm in diameter (Scale bar: 100 nm), **(G)** a size range that agreed with that measured by DLS.

We investigated whether exosomes affect Aβ-induced LTP impairment in the CA1 region of the dorsal hippocampus *in vivo*. We used ADDLs [5] prepared from synthetic Aβ_1-42_ and exosomes isolated from the conditioned media of cultured N2a neuroblastoma cells (Figure 1). On SDS-PAGE, ADDLs yielded 3 bands which migrated with molecular weights of ~4 (monomer), ~12 (trimer) and 16 (tetramer) kDa (Figure 1A). By dynamic light scattering (DLS), ADDLs contained a mixture of species with hydrodynamic radii (R_*H*_) ranging from ~10 to 30 nm (Figure 1B), but by atomic force microscopy (AFM) only small (3 - 6 nm) globular structures were detected (Figure 1C, D). The apparent size discrepancy for the Aβ species present in our ADDL preparation is likely to result from technical limits of the used methods. Specifically, since SDS-PAGE is highly denaturing, it might not be suitable for determination of native sizes of Aβ assembly, but could be used to distinguish the SDS-stable forms from labile Aβ species. While AFM could be used to detect oligomeric forms of Aβ, certain assemblies would not adhere to mica well enough and as a result, were not detected. Nonetheless, our characterization of ADDLs revealed the presence of a heterogeneous mixture of different Aβ species, some of which were at least partially stable in SDS and which existed as small globular structures of 3 - 6 nm [5,19]. To prepare exosome fractions, we had excluded plasma membrane-derived fragments and other non-exosomal vesicles through the optimized procedures [20]. Contrasting to the vesicles originated from Golgi body that float at 1.05 to 1.12 g/ml and endoplasmic reticulum-derived vesicles at 1.18 to 1.25 g/ml, exosomes are the only vesicles sizing 30 ~ 100 nm and gradient density ranging 1.13 ~ 1.19 g/ml (Figure 1E) [12,20]. Exosomes are further defined by their expression of marker proteins such as Flotillin-1, Alix or PrP^C^ that are highly enriched in the exosomal fractions (Figure 1E), their ultrastructure and size (Figure 1F, G) [12]. Altogether, we verified that our procedures were able to yield relatively pure exosomes [12,20].

**Figure 2:**
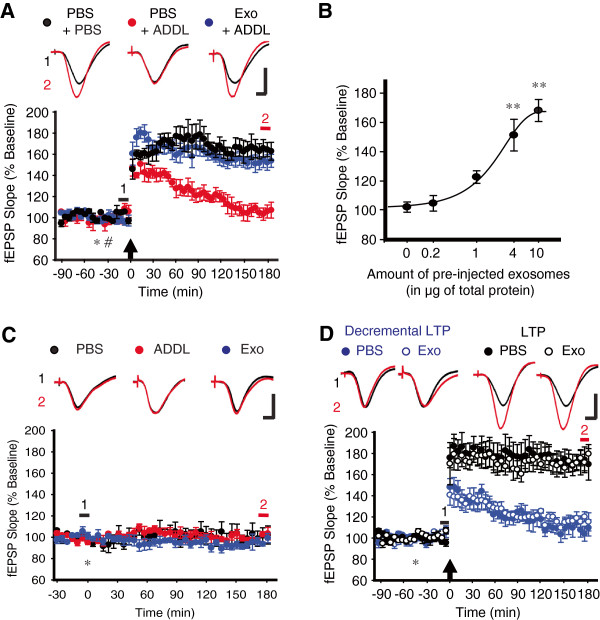
**Exosomes abrogate ADDL-mediated disruption of LTP. (A)** Prior infusion of exosomes (4 μg in 5 μl, asterisk) prevented ADDLs (10 pmol in 5 μl, hash) inhibiting LTP. Animals received sequential injections of exosomes and ADDLs before the application of HFS (arrow). *Insets* show representative traces at the color-matched time points. Calibrations: 1.5 mV and 10 ms for all traces. **(B)** Dose-dependent protective effect of exosomes against ADDL-mediated disruption of LTP (0 μg Exo + ADDL, 100 ± 3%, n = 5; 0.2 μg Exo + ADDL, 105 ± 5%, n = 4; 1 μg Exo + ADDL, 123 ± 5%, n = 4; 4 μg Exo + ADDL, 151 ± 10%, n = 6; 10 μg Exo + ADDL, 168 ± 9%, n = 4). Statistical significance was expressed as **, *P* < 0.01 comparing to control group injected with 0 μg Exo + ADDL. **(C)** Neither exosomes nor ADDLs (5 μl, asterisk) affected baseline excitatory synaptic transmission in the CA1 area *in vivo* (PBS, n = 4; ADDL, n = 5; Exo, n = 4). **(D)** Exosomes (4 μg in 5 μl, asterisk) did not enhance decremental or standard LTP. An arrow indicates the time point of application of either weak HFS for decremental LTP or HFS for standard LTP, respectively. *Insets* and calibrations as in **A**. Error bars, ± SEM.

In agreement with prior reports [5], high-frequency stimulation (HFS) failed to trigger robust LTP in anesthetized rats that had received i.c.v. injection of ADDLs (PBS + ADDL, 105 ± 6%, n = 4 vs. PBS + PBS, 166 ± 10%, n = 4 at 3 h post-HFS, *P* < 0.001, one-way ANOVA with *post hoc* Tukey; Figure 2A). Somewhat unexpectedly, prior infusion of 4 μg exosomes markedly attenuated the synaptic-plasticity-disrupting action of ADDLs. Indeed, despite the administration of ADDLs, HFS now induced robust LTP that was comparable to the control levels and which remained stable for more than 3 h (Exo + ADDL, 152 ± 6%, n = 5, *P* < 0.01 vs. PBS + ADDL; *P* > 0.4 vs. PBS + PBS, one-way ANOVA with *post hoc* Tukey; Figure 2A). Of note, the effect of exosomes against ADDL-induced LTP inhibition was largely dependent upon the amount of exosomes, producing a significant effect when 4 μg or more was infused (Figure 2B). Unless otherwise specified, therefore, we used 4 μg exosomes in the subsequent studies. In this condition, however, neither exosomes nor ADDLs significantly affected baseline synaptic transmission (Figure 2C). Exosomes might exert this protective effect by enhancing LTP *per se*, and/or functionally counteract the plasticity-disrupting effect of ADDLs. When we examined the ability of exosomes to convert decremental LTP into stable LTP or boost control LTP, however, we did not detect any significant difference on weak HFS-induced decremental LTP (PBS, 106 ± 7%, n = 5 vs. Exo, 117 ± 6%, n = 4, *P* > 0.3, unpaired t-test; Figure 2D) or standard HFS-induced LTP (PBS, 172 ± 13%, n = 5 vs. Exo, 175 ± 8%, n = 4, *P* > 0.8, unpaired t-test; Figure 2D). Thus, direct facilitatory effects on the magnitude of LTP are unlikely to account for the capability of exosomes to rapidly abrogate the inhibitory effects of ADDLs.

### ADDLs are sequestered on the surface of exosomes

**Figure 3:**
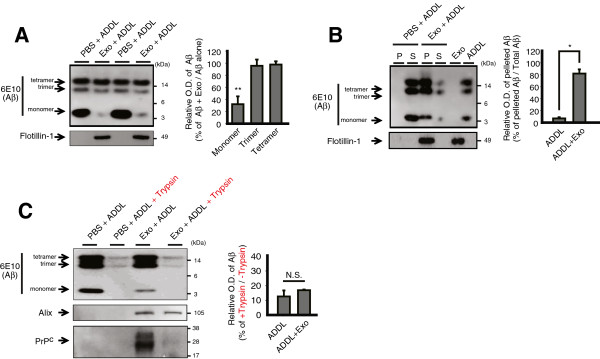
**ADDLs bound to exosomes*. *(A)** Immunoblots of ADDLs after incubation either with PBS or exosomes (left top). Exosomes present in each lane were verified with Flotillin-1 (left bottom). Right, relative optical density (O.D.) of Aβ species. **(B)** Immunoblot results of *in vitro* binding assay of Aβ and exosomes (left top). Either exosomes or ADDLs alone were loaded as controls. Left bottom, exosomes were also verified with an exosomal marker Flotillin-1 (P, pellet; S, supernatant). Right, relative O.D. of pelleted Aβ. **(C)** Exosome-bound ADDLs remained accessible to digestion by trypsin (left top). Limited proteolysis with trypsin resulted in cleavage of exosomal surface protein PrP^C^, but not the intraluminal protein Alix (left, middle and bottom). Very low amount of ADDLs remained after trypsin treatment and the levels of which were similar irrespective of the presence or absence of exosomes (right, relative O.D. of Aβ). Error bars, ± SEM. Statistical significance was expressed as *, *P* < 0.05; **, *P* < 0.01.

To address possible mechanisms underlying the protective action of exosomes against ADDL-induced LTP inhibition, we first examined whether exosomes degrade Aβ, which could abrogate the plasticity-disrupting effect. When we incubated ADDLs with exosomes in the same ratio used for LTP experiments, this resulted in a loss of the Aβ species that migrated at ~4 kDa (monomer) on SDS-PAGE (32 ± 13%, *P* < 0.01, n = 5, Mann-Whitney *U* test; Figure 3A). Unlike Aβ monomer, Aβ oligomers were largely unaffected by the incubation with exosomes (~12 kDa Aβ, 96 ± 10%, *P* > 0.5; ~16 kDa Aβ, 97 ± 5%, *P* > 0.05, n = 5, Mann-Whitney *U* test; Figure 3A), indicating that exosomes did not effectively degrade Aβ oligomers at least over the time course of our experiments. Although the reason for the loss of Aβ monomer is unclear, it could result from the degradation of authentic Aβ monomer by exosomal proteases such as insulin-degrading enzyme (IDE) [15,21]. However, since monomeric Aβ does not inhibit LTP [22] and IDE is not believed to degrade plasticity-disrupting Aβ oligomers [23], such degradation would not be expected to contribute to the rescue of the ADDL-mediated block of LTP.

On the other hand, exosomes might decrease free Aβ oligomers available by shifting free Aβ to the exosome-bound Aβ. We examined this possibility by incubating ADDLs with exosomes and then physically separating (by centrifugation) exosomes from unbound Aβ. A major proportion of Aβ oligomers co-migrated with the exosomes that were readily pelleted with ultracentrifugation whereas only a small fraction of free ADDLs remained in the supernatant fraction (mean % of pelleted Aβ relative to total Aβ: PBS + ADDL, 7 ± 2% vs. Exo + ADDL, 82 ± 7%, *P* < 0.05, n = 3, Mann-Whitney *U* test; Figure 3B). However, this could have resulted potentially from Aβ assemblies that were simply pelleted to the exosome-containing fraction after being aggregated by exosomes [16], rather than being directly bound to exosomes. Therefore, we have corroborated the direct binding of Aβ assemblies and exosomes by directly pulling down the exosome-bound Aβ after their *in vitro* incubation (Additional file 1: Figure S1), which argues against the possibility.

To elucidate the possible fate of Aβ oligomers following binding onto exosomes, we have developed a partial trypsinization protocol to degrade only proteins on the outside of exosomes (see Methods for detailed information) and applied this method after the incubation of exosomes and ADDLs. If ADDLs were internalized into exosomes, the resultant ADDLs residing in the lumen of exosomes should be resistant to trypsin, which would likely leave more ADDLs remaining after the treatment of trypsin. Inconsistent with this notion, the remaining amount of ADDLs did not differ in the absence and presence of exosomes (% of remaining Aβ after trypsinization: PBS + ADDL, 12 ± 4% vs. Exo + ADDL, 17 ± 1%; *P* = 0.51, n = 3, Mann-Whitney *U* test; Figure 3C). Therefore, a major proportion of ADDLs remains on the surface of exosomes even after binding to exosomes in the time frame we examined, rather than being internalized into exosomes. Collectively, it is reasonable to speculate that the protective effect of exosomes against ADDL-induced LTP impairment arises from sequestering and immobilization of Aβ oligomers at the surface of exosomes.

### Exosomal surface proteins including PrP^C^ are required for the protective role of exosomes against Aβ

**Figure 4:**
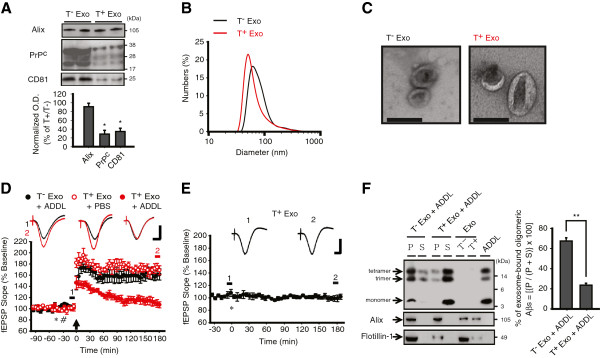
**Exosomal surface proteins are essential for the protective role of exosomes against the synaptic-plasticity-disrupting action of ADDLs. (A)** Trypsinization (T^+^) was effective in removing surface proteins (PrP^C^ and CD81), but not luminal protein (Alix) of N2a cell-derived exosomes when compared to non-trypsinized (T^-^) control. Top, representative blots and bottom, normalized mean O.D. of immunoblots, n = 5. **(B)** Trypsinized exosomes (T^+^ Exo, red line) had similar sizes (74 ± 3 nm), compared with those of non-trypsinized exosomes (T^-^ Exo, black line) (83 ± 7 nm, *P* > 0.05, n = 3), as measured by DLS. **(C)** By EM, the structure and size of T^+^ Exo were indistinguishable from T^-^ Exo (Scale bar: 100 nm). **(D)** T^+^ Exo (5 μl, asterisk) was unable to prevent the plasticity-disrupting-activity of ADDLs (10 pmol in 5 μl, hash), but which can be neutralized by T^-^ Exo. An arrow indicates HFS application and *insets* show representative traces at the color-matched time points. Calibration: 1.5 mV and 10 ms. **(E)** Lack of effect of i.c.v. injection (asterisk) of T^+^ Exo (n = 4) on the stability of fEPSP recordings. **(F)** Comparing to T^-^ Exo, T^+^ Exo showed a reduced ability to sequester ADDLs. Left panel: representative immunoblot using antibodies against Aβ (6E10), Alix and Flotillin-1 (P, pellet; S, supernatant). Right panel: mean % of ~12 and 16 kDa Aβ bound to exosomes (P) relative to total ~12 and 16 kDa Aβ (P + S). Error bars, ± SEM. Statistical significance was expressed as *, *P* < 0.05; **, *P* < 0.01.

To further investigate the direct interaction of ADDLs with exosomes, we used trypsin in a mild condition (see Methods for details) to assess whether exosomal surface proteins were required for ADDL-neutralizing activity. The partial trypsinization efficiently removed the exosomal surface proteins while leaving the luminal protein intact (Figure 4A), and without affecting the integrity of exosomes (Figure 4B, C). Importantly, the trypsinized exosomes were no longer capable of rescuing the ADDL-mediated block of LTP (T^-^ Exo + ADDL, 161 ± 9%, n = 5 vs. T^+^ Exo + ADDL, 107 ± 6%, n = 4, *P* < 0.01, one-way ANOVA with *post hoc* Tukey; Figure 4D), and did not alter either LTP *per se* (T^+^ Exo + PBS, 170 ± 7%, n = 4, *P >* 0.9 compared to PBS + PBS, one-way ANOVA with *post hoc* Tukey; Figure 4D) or baseline synaptic transmission (Figure 4E). In agreement with the LTP results, trypsinized exosomes bound only a smaller fraction of Aβ oligomers compared to non-trypsinized exosomes (T^-^ Exo + ADDL, 67 ± 3% vs. T^+^ Exo + ADDL, 24 ± 2%, *P* < 0.01, n = 5, Mann-Whitney *U* test; Figure 4F). These data indicate that surface proteins of exosomes are required for the sequestration of synaptotoxic Aβ assemblies, which is consistent with prior reports that binding of Aβ oligomers to neuronal membranes is mediated by trypsin-sensitive molecules [5].

**Figure 5:**
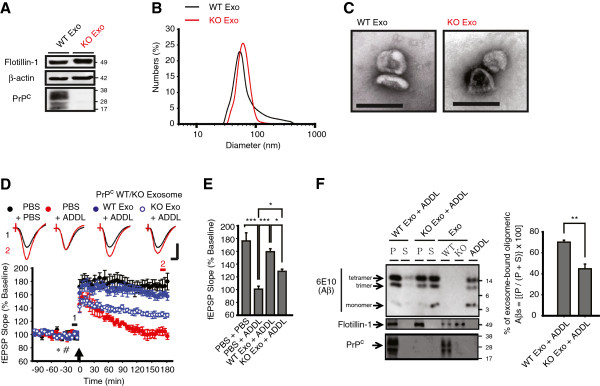
**PrP**^**C **^**on the surface of exosomes is essential for ADDL-neutralizing activity of exosomes. (A)** Exosomes prepared from PrP^C^ WT or KO cells both revealed Flotillin-1 and β-actin, but PrP^C^ was absent in KO exosomes. **(B)** Exosomes lacking PrP^C^ were similar in size (KO Exo, red line: 87 ± 4 nm) to WT exosomes (WT Exo, black line: 84 ± 19 nm, *P* > 0.05, n = 3). **(C)** By EM, the structure of WT Exo and KO Exo was indistinguishable (Scale bar: 100 nm). **(D)** KO Exo only partially protected against the ADDL-mediated inhibition of LTP compared to WT Exo. An equivalent amount of exosomes (4 μg in 5 μl, asterisk) or ADDLs (10 pmol in 5 μl, hash) was injected in each designated condition. An arrow indicates HFS application and *insets* show representative traces at the color-matched time points. Calibration: 1.5 mV and 10 ms. **(E)** A summary histogram of the data in **D** with statistical comparisons. **(F)** KO Exo exhibited reduced ADDL-sequestering activity when compared to WT Exo. Left panel: representative immunoblots using antibodies against Aβ (6E10) or Flotillin-1 as well as PrP^C^. Right panel: mean % of ~12 and 16 kDa Aβ bound to exosomes (P) relative to total ~12 and 16 kDa Aβ (P + S). Error bars, ± SEM. Statistical significance was expressed as *, *P* < 0.05; **, *P* < 0.01; *** *P* < 0.001.

Aβ oligomers bind to PrP^C^, a cell membrane-bound glycoprotein that express abundantly in the central nervous system, specifically and with high affinity [6,7,24]; PrP^C^ was also known to be expressed at high levels on exosomes [25,26]. Thus, we sought to examine whether exosomal PrP^C^ contributes to the sequestration of ADDLs by exosomes. To this end, we prepared exosomes from either *Prnp*^*+/+*^ (wild-type, PrP^C^ WT) or *Prnp*^*-/-*^ (PrP^C^ knock-out, PrP^C^ KO) hippocampal cell lines [27] (Figure 5A-C). Because we injected exogenously PrP^C^ WT or KO exosomes through i.c.v. to wild type rat throughout the study, acute infusion of these exosomes into the brain can change only PrP^C^ of extracellular exosomes alone, but not neuronal PrP^C^ level of subject animals. Intriguingly, the effectiveness of PrP^C^ KO exosomes in preventing ADDL-induced LTP inhibition was significantly reduced compared with that of WT exosomes (WT Exo + ADDL, 159 ± 5%, n = 6, *P* < 0.001 vs. PBS + ADDL, 101 ± 5%, n = 6; *P* < 0.05 vs. KO Exo + ADDL, 129 ± 3%, n = 5, one-way ANOVA with *post hoc* Tukey; Figure 5D, E) and the binding of Aβ oligomers to PrP^C^ KO exosomes was also significantly decreased compared to WT exosomes (WT Exo + ADDL, 70 ± 2% vs. KO Exo + ADDL, 45 ± 5%; *P* < 0.01, n = 5, Mann-Whitney *U* test; Figure 5F). The finding that knock-out of exosomal PrP^C^ reduced ADDL binding and the exosome-induced rescue of LTP to a similar extent suggests that both effects are mediated, at least in part, through PrP^C^.

### Both N2a cell- and human CSF-derived exosomes prevent AD brain-derived Aβ from affecting LTP

**Figure 6:**
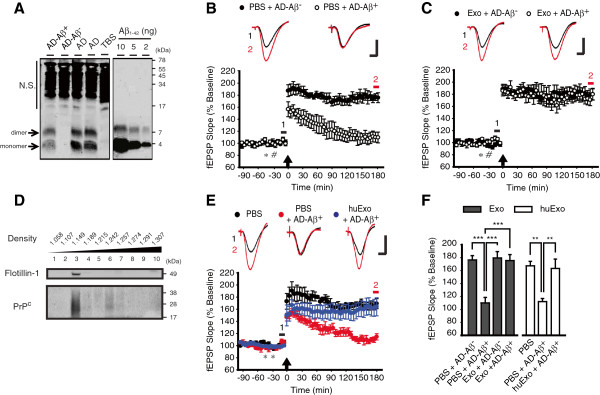
**Both N2a cell- and human CSF-derived exosomes prevent AD brain-derived Aβ from inhibiting *in vivo *LTP. (A)** Immunoprecipitation/Western blot analysis of TBS extracts of AD brain. Comparing to synthetic Aβ_1-42_ loaded for control, AD-Aβ^+^ contained 2.2 ng/ml of Aβ monomer and 0.76 ng/ml of Aβ dimer whereas Aβ was completely removed from AD-Aβ^-^. AD stands for the starting AD brain extract, TBS for buffer vehicle and N.S. denotes non-specific bands, presumably that arise due to the reaction of the IP antibody with the antibodies used for Western blotting. **(B)** Infusion of AD-Aβ^+^ disrupted LTP *in vivo*, but not by AD-Aβ^-^. Animals were pre-injected with PBS (5μl, asterisk) followed by a second injection (hash) of either AD-Aβ^-^ or AD-Aβ^+^ containing ~18 pg Aβ. An arrow indicates HFS application and *insets* show representative traces at the color-matched time points. Calibration: 1.5 mV and 10 ms. **(C)** Cell-derived exosomes prevented AD-Aβ^+^ from disrupting LTP. In animals that received N2a cell-derived exosomes (4 μg in 5 μl, asterisk), AD-Aβ^+^ (6 μl, hash) no longer inhibited LTP, similar to animals injected with AD-Aβ^-^ (6 μl, hash). Arrow, *insets* and calibration as in **B**. **(D)** The exosomes prepared from human CSF were detected in fractions with a buoyant density around 1.149 g/ml and contained Flotillin-1 and PrP^C^. **(E)** Infusion of human CSF-derived exosomes (huExo) abrogated the disruption of LTP by AD-Aβ^+^. Administration (asterisks, total 10 μl i.c.v., spread over two infusions) of samples of AD-Aβ^+^ extracts after 30 min pre-incubation with CSF exosomes (total 1 μg) no longer inhibited LTP. Arrow, *insets* and calibration as in **B**. **(F)** A summary histogram of the data in **B**, **C** and **E** with statistical comparisons. Error bars, ± SEM. Statistical significance was expressed as **, *P* < 0.01; ***, *P* < 0.001 comparing to respective control.

Because it remains unknown whether Aβ assemblies formed *in vitro* accurately represent Aβ species found in human brain, we investigated if exosomes could prevent the disruptive activity of the most disease-relevant form of Aβ, Aβ extracted from AD brain. Aqueous extracts of AD brain contained Aβ species which migrated on SDS-PAGE as monomers and dimers (Figure 6A) and potently inhibited LTP (Figure 6B, E and F). Consistent with our previous reports [11], this inhibition of LTP was attributable to Aβ but not any other components of the AD extract since specific removal of Aβ reversed this effect, whereas mock-immunodepletion did not (PBS + AD-Aβ^+^, 110 ± 9%, n = 5 vs. PBS + AD-Aβ^-^, 176 ± 7%, n = 5, *P* < 0.001, one-way ANOVA with *post hoc* Tukey; Figure 6B, F). Importantly, the i.c.v. infusion of N2a cell-derived exosomes fully abrogated the inhibitory effect of Aβ-containing AD brain extracts (Exo + AD-Aβ^+^, 175 ± 9%, n = 7, *P* < 0.001 vs. PBS + AD-Aβ^+^, one-way ANOVA with *post hoc* Tukey; Figure 6B, C and F), but did not affect LTP induced in the presence of Aβ-immunodepleted AD brain extracts (Exo + AD-Aβ^-^, 179 ± 10%, n = 5, *P* > 0.05 vs. PBS + AD-Aβ^-^, one-way ANOVA with *post hoc* Tukey; Figure 6B, C and F).

Next, we tested whether human brain-derived exosomes could also neutralize plasticity-disrupting forms of Aβ. To do this, we isolated exosomes from the CSF of healthy volunteers **(**Figure 6D). Due to the limited amount of CSF exosomes available, we modified our experimental paradigm to pre-incubating CSF exosomes with AD brain extracts and then injecting the mixture before HFS as previously used [10]. When CSF exosomes (1 μg) were pre-incubated with AD brain extracts, normal LTP was induced whereas injection of the same Aβ-containing AD brain extracts without exosomes consistently inhibited LTP (huExo + AD-Aβ^+^, 163 ± 14%, n = 5, *P* > 0.9 vs. PBS, 167 ± 7%, n = 4; *P* < 0.01 vs. PBS + AD-Aβ^+^, 112 ± 5%, n = 5, one-way ANOVA with *post hoc* Tukey; Figure 6E, F). These results demonstrate that CSF-derived exosomes can also protect LTP against the plasticity-disrupting activity of AD brain-derived Aβ.

## Discussion

Although cellular functions of exosomes in nervous system are not completely understood, previous studies have provided evidence that exosomes can participate in paracrine delivery of biologically active and infectious materials such as Aβ, PrP^C^ and α-synuclein [28-31]. It was also suggested that lipid components or IDE on the surface of exosomes were involved in the regulation of Aβ by activating fibrillization or proteolysis [15,16,32,33]. Although those reports had suggested the potential interaction between Aβ and exosomes, however, the physiological role of exosomes remains largely unknown particularly for Aβ-induced synaptotoxicity [15,16,18]. This would stem from the fact that the controlled manipulation of the levels of exosomes in the brain is very difficult and thus the direct assessment of the putative roles that exosomes exert has been hampered. Using an infusion paradigm, we discovered that addition of exogenous exosomes into brain can abrogate the synaptic-plasticity-disrupting activities of Aβ, most likely through direct sequestration of Aβ oligomers.

Whereas it is generally postulated that synaptic failure in AD is caused by soluble Aβ assemblies, the molecular mechanisms whereby Aβ assemblies are formed and maintained for AD pathogenesis remains unclear yet [4]. Although we could not fully identify the molecular identity of synaptotoxic Aβ assemblies, we confirmed that our Aβ preparations from synthetic Aβ_1-42_ peptide or AD brain extraction can effectively inhibit synaptic plasticity *in vivo*[5,6,11,34]*,* which validated the efficacy of the used experimental conditions. Furthermore, multiple characterization assays that we used revealed the presence of a heterogeneous mixture of different sized Aβ species and also relatively pure exosomal preparation, consistent with previous reports [5,12,19,20]. In addition to the protective effect of exosomes against synaptotoxic activity of ADDLs, we found that exosomes were able to ameliorate the plasticity-disrupting activity of the most pathophysiologically-relevant form of Aβ, Aβ extracted from AD brain. Interestingly, we had to inject at least 45 ng of ADDLs and only 18 pg of Aβ from AD brain to produce potent inhibition of LTP. We and others have previously reported that the potency of synthetic Aβ used to disrupt the memory of learned behavior or to impair LTP is usually several orders of magnitude higher than that of naturally produced Aβ from the AD brain or APP expressing-cultured cell lines [11,34,35]. The different potency of the two Aβ preparations used in the present experiments likely reflects the fact that although they contained similar concentrations of synaptic plasticity disrupting Aβ, other additional assemblies, that are presumed to be relatively inactive, are present in higher concentration in the ADDL preparation compared to AD brain extracts.

The protective effect of exosomes against the synaptic-plasticity-disrupting activity of Aβ leaves the question about the underlying mechanisms. As Aβ itself is a sticky protein and exosomes contain a variety of proteins and lipid components, there are several possibilities including non-specific proteolysis or sequestration such that a number of proteins, lipids, and membranous vesicles could affect Aβ-mediated LTP inhibition. Throughout biochemical experiments and *in vivo* electrophysiology, however, we demonstrate that proteolysis of Aβ is unlikely account for the protective effect; rather exosomes could sequester Aβ oligomer in a manner analogous to binding of neutralizing antibodies [10,36]. Still, we cannot directly interpret the effect of decreased Aβ monomer by exosomes *in vivo* since the effect of monomeric Aβ on synaptic plasticity had been examined only on hippocampal slice and the different protocol used to induce LTP could also compound Aβ-induced synaptic alteration [22,37]. Over brain slices and primarily cultured neurons, Aβ monomer showed protective effects on LTP and neuronal survival [38,39]. Therefore, the possible outcomes that chronically-decreased Aβ monomer produces should be further studied. Moreover, the sequestration of Aβ depends upon surface proteins of exosomes such as PrP^C^, which supports the idea that specific involvement of exosomal surface proteins to capture or immobilize soluble Aβ oligomer at exosomes. Especially, the ineffectiveness of trypsinized exosomes in neutralizing synaptotoxicity of Aβ argues against the possibility of involving exosomal lipids for the exosomes’ protective effect. Because we examined only the effect of exosomes in this study, the effect of other small membranous vesicles that also derive from plasma membrane or other intracellular origins on Aβ-mediated synaptotoxicity should be verified further.

The role of PrP^C^ as a putative receptor for Aβ oligomer, and its involvement in Aβ-mediated impairment of LTP has been intensely debated [7,40-42], but there is no controversy regarding the ability of PrP^C^ to bind Aβ. Multiple independent studies concluded that PrP^C^ binds Aβ oligomers specifically and with high affinity [6,7,40,43-45]. In this study, we detected that exosomes from PrP^C^-deficient cells are significantly less able to protect against Aβ than PrP^C^-containing exosomes. This PrP^C^-dependent effect of exosomes would be due to the high affinity binding of Aβ to PrP^C^. Therefore, exosomes that derived from cell lines expressing mutated PrP^C^ at the binding site for Aβ (95-105 residues of PrP^C^) could be used to further verify the function of PrP^C^ in detail [7,45]. In this study, we can use only the exosomes from “immortalized cell lines” of WT or PrP^C^-depleted neurons due to the difficulty to collect large amount of exosomes from primarily cultured neuron or from CSF of genetically modified mice. Notably, the fact that ablation of PrP^C^ did not completely obviate the protective effects of exosomes suggests that exosomal proteins other than PrP^C^ might also contribute to the sequestration of toxic Aβ oligomers, as consistent with previous observation that Aβ binding was only partially reduced to PrP^C^-deficient neurons [7]. To elucidate the full repertoire of candidate exosomal proteins involved in the interaction with Aβ and to further understand their molecular mechanisms, further screening and functional studies will be necessary.

It might be informative to examine whether exosomes in culture medium play a protective role against the toxic effect of Aβ on primarily cultured neuron. However, Aβ-induced deficit in synaptic plasticity normally occurs well before manifest loss of neurons in AD models [2,4,46]. To establish whether the effects of Aβ and exosomes on synaptic plasticity are reflected at the level of cognition, behavioral tests determining their effect on cognitive function will be required.

In this study, we provide evidence for the neutralizing action of exosomes against Aβ-induced LTP impairment using both N2a cell-derived exosomes and human CSF-derived exosomes. These observations raised an important question: Do endogenous exosomes normally prevent Aβ-mediated impairment of synaptic plasticity? However, demonstration for effects of endogenous exosomes on AD pathogenesis or Aβ-induced alteration of synaptic plasticity was very challenging due to the difficulties to modify the nature and quantity of exosomes in the brain without any side effects. For example, when we activated the recycling of endosomes to increase the release of exosomes, the manipulation might affect production and release of Aβ [47]. The amount of exosomes prepared from human CSF or interstitial fluid of brain has been measured only in a few studies for its scarcity [48]. Although we were also unable to quantify the exosome content in a systematic manner due to the limited supply of fresh CSF, we did obtain approximately 8 μg of exosomes from 10 ml of human CSF following the purification steps including density gradient fractionation that normally involves considerable loss of exosomes (up to 60 % of the starting amount; see ref. Tauro et al. [49]). Accordingly, we estimated ~ 2 μg endogenous exosomes contained in 1 ml of human CSF as consistence with previous study [48]. Therefore, we surmise that our i.c.v. infusion of 4 μg exosomes would yield ~ 4 times the concentration of endogenous exosomes present in rat CSF, assuming that rat CSF (ranged 500 μl total; see ref. Lai et al. [50]) contained an exosome content similar to that of human CSF. Importantly, 1 μg of CSF-derived exosomes exhibited a significantly protective effect when co-injected with Aβ-containing AD brain extracts (Figure 6). Taken together, we speculate that exosomes may protect synaptic plasticity against amyloidogenic insults *in situ* particularly over an extended time window. Considering studies indicating the increased release of exosomes by 2.5 - 4 folds under *in vitro* hypoxia condition [15,51], it is very likely that this process could be occurred in pathological condition. Eventually, exosome-bound Aβ might be taken up by microglia for degradation in normal condition [16], or they can be the seed for the plaque formation in pathological condition [52]. The efficiency of this process may be critical in determining the onset and progression of AD given the causal contribution of synaptic failure to the disease and cognitive decline [2]. Since both Aβ and exosomes are released from the brain in an activity-dependent manner [53,54], the dynamic change of exosome concentration in brain, especially in AD patients, is a subject that we feel should be explored further.

## Conclusions

Collectively, exosomes are able to sequester synaptotoxic Aβ oligomers via surface proteins such as PrP^C^ and thereby rescue LTP from Aβ-mediated impairment *in vivo*. Importantly, our findings based on exosomes isolated from human CSF and Aβ from AD brain strongly indicate that the pathophysiologically relevant forms of Aβ in the brain can be sequestered by exosomes. Although we were unable to quantitatively measure the change of total exosome concentration in the brain after exogenous application, at the very least we were successful in using human CSF samples to provide a reasonable and predictive window on exosome levels in the brain and thus to assess the utility of this measure as a biomarker for AD. Similarly, when we can manipulate the levels of endogenous exosomes in a more precise manner, we will be in a better position to ascertain their pathophysiological contribution to AD and perhaps supply exogenous exosomes or artificially engineered forms of lipid vesicles for a therapeutic benefit.

## Methods

### Animals

Male Wistar rats (250 - 350 g) were used for *in vivo* recording experiments. They were housed under a 12-hour light/dark cycle and given *ad libitum* access to food and water. The rats were anesthetized with urethane (ethyl carbamate, 1.5 g/kg, *i.p.*). The body temperature was maintained at 37.4 - 38°C for the duration of the experiments. All procedures for animal experiments were approved by the ethical review committee of Trinity College Dublin and the Department of Health and Children, Ireland and POSTECH (Pohang University of Science & Technology), Korea and performed in accordance with the relevant guidelines.

### ADDLs preparation

Aβ_1-42_ (American peptide) was dissolved in 1,1,1,3,3,3-hexafluoro-2-propanol (Sigma) to a concentration of 1 mM. The solution was allowed to evaporate for 2 h and then dried in a Speed Vac. The resulting film of peptide was stored at -20°C or immediately resuspended in dimethyl sulfoxide (Sigma) to produce a 1 mM solution. This solution was sonicated for 10 min in a sonic bath, and then diluted to 100 μM in phenol red-free Ham’s F12 medium (Life Technology) and incubated for 12 h at 4°C. The resulting solution was then spun at 100,000 g for 1 h and either used immediately or stored at -80°C for up to 2 weeks. Monomeric Aβ_1-42_ was prepared by dissolving the peptide film to 100 μM in 10 mM NaOH solution (pH 11).

### Isolation of exosomes

N2a cells were grown in exosome-depleted medium comprising 44.5% DMEM, 44.5% Opti-MEM with 10% FBS and 1% Penicillin/Streptomycin under a humidified environment of 5% CO_2_/95% O_2_ incubator at 37°C. PrP^C^ WT or KO cells (HW8-1 and Hpl3-4, respectively) established from the primary cultured hippocampal neuron of *Prnp*^*+/+*^ and *Prnp*^*-/-*^ mice [27] were grown in exosome-depleted medium composed of 89% DMEM, 10% FBS and 1% Penicillin/Streptomycin. Exosomes were prepared as previously described [20] with minor modifications. In brief, exosome-enriched media was fractionated by centrifugation (200,000 g × 2 h) on a 5 - 30% of opti-prep gradient (Axis-Shield) in a SW-41 rotor (Beckman Coulter). 1 ml from each fraction was collected and diluted 1:10 with pre-cooled phosphate-buffered saline (PBS) and collected by centrifugation for 1 h at 100,000 g. A portion of the resultant pellets were boiled in 2× sample buffer and used for Western blotting. Fractions enriched in exosomes were used for subsequent studies. The amount of exosomes used was expressed in terms of total protein which was determined using the Pierce BCA assay kit (Thermo Scientific).

All procedures for collection and usage of human CSF were approved by the Mater Misericordiae University Hospital Research Ethics committee, Ireland. CSF was obtained from a 61-year-old female and a 71-year-old male donors both of whom were healthy and cognitively normal. 10 ml of CSF in total was taken by lumbar puncture from the L3/L4 interspace, and kept on ice. CSF was used to isolate exosomes within 2 h of collection, using the procedure described above.

### Western blotting

Samples containing Aβ were mixed with 4X NuPAGE® LDS sample buffer and electrophoresed on NuPAGE® 4 - 12% Bis-Tris gels (Life Technology). Proteins were transferred onto PVDF membrane (Millipore) and the membrane blocked using 5% skim-milk solution was immunoblotted with the anti-Aβ antibody, 6E10 (Covance). For detection of exosomal proteins, samples were boiled after being mixed with 5X sample buffer, then electrophoresed on 10% polyacrylamide SDS gels, transferred onto PVDF membrane and finally immunoblotted with antibodies against Alix (BD Bioscience), Flotillin-1 (BD Bioscience), PrP^C^ (ICSM-35, D-Gen), CD81 (Santa Cruz Biotechnology) or β-actin (Sigma). Immunoreactive bands were visualized using horseradish peroxidase-conjugated goat anti-mouse IgG secondary antibody (1:3000) (Signalway Antibody) and images were collected by Las-4000 (Fujifilm Life Science). The western blot images were analyzed using Image J software (NIH).

### Atomic force microscopy (AFM)

10 μl of 10 μM ADDLs in PBS was incubated on freshly cleaved mica for 1 min. The mica was washed twice with 100 μl of deionized water and dried under a gentle stream of N_2_ gas. Tapping mode AFM imaging was performed in air using Multimode/Nanoscope IIIa (Digital instruments) equipped with a J-scanner. The images were taken with a TESP cantilever (Veeco) at a sample rate of 0.85 Hz. Section analysis (Nanoscope V) was employed to measure the z-height of distinct globules (>50) and the z-height was used as a representative value for the size of Aβ oligomers [19].

### Electron microscopy (EM)

5 μl drops of exosomes (50 μg/ml) were loaded onto carbon-coated 200 μm copper grids and incubated for 1 min. The samples were then stained with 2% uranyl acetate for 2 min, and excess solution carefully removed and the grid left to air dry. Images were captured using an electron microscope (JEOL) operated at 100 kV.

### Dynamic light scattering (DLS) spectroscopy

The sizes of exosomes (10 μg in 100 μl) or ADDLs (10 μM in 100 μl) were measured by DLS performed with Zetasizer Nanoseries instrument (Malvern Nano-Zetasizer). The mean values of particle sizes were obtained from more than 3 independent preparations.

### *in vivo* electrophysiology and i.c.v. infusion

Electrodes were made and implanted in anaesthetized animals as described previously [6]. Briefly, twisted-wire bipolar electrodes were constructed with Teflon-coated tungsten wires (62.5 μm inner core diameter, 75 μm external diameter, A-M Systems). Field excitatory postsynaptic potentials (fEPSPs) were recorded from the stratum radiatum of the CA1 area of the right dorsal hippocampus in response to stimulation of the ipsilateral Schaffer collateral-commissural pathway. Electrode implantation sites were identified using stereotaxic coordinates relative to bregma, with the recording site located 3.4 mm posterior to bregma and 2.5 mm right of midline, and the stimulating electrode located 4.2 mm posterior to bregma and 3.8 mm right of midline. The optimal depth of the electrodes was determined using electrophysiological criteria and verified post-mortem. Test fEPSPs were evoked at a frequency of 0.033 Hz at the stimulation intensities adjusted to elicit fEPSP amplitudes of 40 - 50% of maximum. The high-frequency stimulation (HFS) protocol for inducing LTP consisted of 10 bursts of 20 stimuli with an inter-stimulus interval of 5 ms (200 Hz), and an inter-burst interval of 2 sec. The intensity was increased so as to give 75% of maximum amplitudes of fEPSPs during the HFS. The weak HFS consisted of 10 bursts of 10 stimuli with an inter-stimulus interval of 10 ms (100 Hz), and an inter-burst interval of 2 sec. The initial slopes of fEPSPs were measured and the average of ten sweeps was plotted. Unless otherwise specified, fEPSP slopes (% Baseline) indicate the mean slopes between 170 - 180 min after HFS in each condition. To infuse samples, a stainless-steel guide cannula (22 gauge, 0.7 mm outer diameter, 13 mm length) was implanted above the right lateral ventricle (1 mm lateral to the midline and 4 mm below the surface of the dura) just prior to electrode implantation. The placement of the cannula was verified post-mortem with i.c.v. infusion of Indian Blue ink dye.

### Binding assays between ADDLs and exosomes

ADDLs were centrifuged for 1 h at 100,000 g prior to incubation with exosomes. The supernatant contained more than 95% of the starting peptide. 1 μg of this ADDL supernatant was then added to identical volumes of purified trypsinized or mock-trypsinized exosomes (160 μg) and incubated at 37°C for 30 min in 10 ml PBS. Thereafter, exosomes were separated from the unbound Aβ by centrifuging for 1 h at 100,000 g. The exosome pellet was dissolved in 2× sample buffer and 25% of the mixture used for Western blotting for exosome-bound Aβ. 25% of the supernatant resulted from 100,000 g centrifugation was collected and used for immunoprecipitation with 6E10/Western blotting for exosome-unbound Aβ. Mean % of ~12 and 16 kDa Aβ bound to exosomes (P) relative to total ~12 and 16 kDa Aβ (P + S) was used for the presentation with bar graph.

### Limited trypsinization for surface proteins of exosomes

Exosomes (0.5 mg/ml) were incubated with trypsin (1 mg/ml, Sigma) for 30 min at 37°C and the reaction was stopped by addition of a serine protease inhibitor Pefabloc SC™ (4 mg/ml, Sigma). After this treatment, exosomes were re-isolated by density gradient centrifugation (as described in the procedures for exosomes isolation). The effect of trypsin on surface and luminal proteins was verified by assessment of trypsinized- and mock-trypsinized exosomes with antibodies against PrP^C^, CD81 (exosomal surface proteins) or Alix (luminal protein).

### Immunoprecipitation of exosomes

Exosomes were incubated with anti-Flotillin-1 antibody (8 μl) and pre-washed Protein A/G agarose bead (Calbiochem) at 4°C for 6 h. The resulting precipitates were washed with PBS and 25% of each sample was used for western blotting.

### AD brain extracts

Human tissue was obtained and used in accordance with local IRB guidelines. A sample of temporal cortex from a 92-year-old woman with a history of dementia and confirmed AD pathology was used to prepare water-soluble extracts and the extracts were examined for the presence of Aβ as described previously [6]. Briefly, a ~2 g cube of frozen tissue was thawed on ice, gray matter isolated, chopped into small pieces with a razor blade and then homogenized in 5 volumes of ice-cold 20 mM Tris-HCl, pH 7.4, containing 150 mM NaCl (TBS) with 25 strokes of a Dounce homogenizer (Fisher). The water-soluble fraction was separated from the insoluble fraction by centrifugation at 91,000 g and 4°C in a TLA 55 rotor (Beckman Coulter) for 78 min and the supernatant was used for the subsequent studies. To eliminate low-molecular-weight bioactive molecules and drugs, the supernatant was exchanged into sterile 50 mM ammonium acetate, pH 8.5 using a 5 ml Hi-trap desalting column (GE Healthcare Bio-Sciences). Thereafter the extracts were divided into 2 parts: one aliquot was immunodepleted (AD-Aβ^-^) of Aβ by 3 rounds of 12 h incubations with the anti-Aβ antibody, AW7 [55], and protein A at 4°C. The second portion was treated identically, but pre-immune serum was used instead of AW7 anti-Aβ antiserum and so produced a “mock”-immunodepleted samples (AD-Aβ^+^). The amount and form of Aβ was analyzed in duplicate 0.3 ml samples by immunoprecipitated with AW7 at a dilution of 1:80 and by western blotting using a combination of the C-terminal monoclonal antibodies, 2G3 and 21F12 (each at a concentration of 1 μg/ml). Detection was achieved using fluorochrome-coupled anti-mouse secondary antibody (1:2500) (Rockland). Images were collected by scanning at 800 nm at a resolution of 169 μm using a Li-COR Odyssey near infrared imaging system (Li-COR Biosciences). Aβ present in the immunoprecipitates were quantified by references of known amounts of synthetic Aβ_1-42_ loadings (2, 5, 10 ng per well) [55].

### Statistical analysis

LTP was expressed as mean ± SEM% of baseline slopes of fEPSPs recorded over at least a 30 min baseline period. Statistical comparisons used paired Student t-tests to compare within single groups of animals for baseline or LTP results whereas unpaired t-tests were employed to compare the LTP between two groups. In the case of multiple comparisons, one-way ANOVA with *post hoc* Tukey test was used. The results from dynamic light scattering or immunoblots were expressed as mean ± SEM % and compared with Mann-Whitney *U* test. Statistical significance between groups is expressed as N.S., not significant; *, *P* < 0.05; **, *P* < 0.01; or ***, *P* < 0.001.

## Abbreviations

AD: Alzheimer’s disease; Aβ: Amyloid β-protein; LTP: Long-term potentiation; ADDL: Aβ-derived diffusible ligand; PrPC: Cellular prion proteins; i.c.v.: *Intracerebroventricularly*; CSF: Cerebrospinal fluid; DLS: Dynamic light scattering; RH: Hydrodynamic radii; AFM: Atomic force microscopy; HFS: High-frequency stimulation; IDE: Insulin degrading enzyme; WT: Wild-type; KO: Knock-out; PBS: Phosphate-buffered saline; EM: Electron microscopy; fEPSP: Field excitatory postsynaptic potential.

## Competing interests

The authors declare that they have no competing interests.

## Authors’ contributions

KA, MJR and J-HK conceived the study. KA, MJR, DMW and J-HK designed the research. KA and IK performed and analyzed *in vivo* electrophysiology experiments. KA, IK, YK, JHJ, GMF, JWP and T-WK performed and analyzed *in vitro* experiments with ADDLs and exosomes. IK, AJM, DK, STO, TL and CAL isolated and characterized human brain extracts and CSF. KA, DMW, MJR and J-HK wrote the paper. All authors read and approved the final manuscript.

## Supplementary Material

Additional file 1: Figure S1 Exosomes neutralize synaptic-plasticity-disrupting activity of Aβ assemblies *in vivo.* Click here for file
