# Supplementary material for: Exosomes neutralize synaptic-plasticity-disrupting activity of Aβ assemblies in vivo
Source: Mol Brain. 2013 Nov 13;6:47. doi: 10.1186/1756-6606-6-47 (PMC4222117; doi:10.1186/1756-6606-6-47)
Supplement: Additional file 1: Figure S1 — Exosomes neutralize synaptic-plasticity-disrupting activity of Aβ assemblies in vivo. [file 1756-6606-6-47-S1.docx]

**Supplemental Information for**

**Exosomes neutralize synaptic-plasticity-disrupting activity of Aβ assemblies *in vivo***

Kyongman An, Igor Klyubin, Youngkyu Kim, Jung Hoon Jung, Alexandra J. Mably, Sean T. O’Dowd, Timothy Lynch, Daniel Kanmert, Cynthia A. Lemere, Gina M. Finan, Yong Song Gho, Joon Won Park, Tae-Wan Kim, Dominic M. Walsh, Michael J. Rowan and Joung-Hun Kim

**Supplemental Method**

**Supplemental Figure 1 and Legend**

**Supplemental Method**

**Immunoprecipitation of exosomes**

Exosomes were incubated with anti-Flotillin-1 antibody (8 μl) and pre-washed Protein A/G agarose bead (Calbiochem) at 4 ^o^C for 6 h. The resulting precipitates were washed with PBS and 25% of each sample was used for western blotting.

**Supplemental Figures 1 and Legend**

**
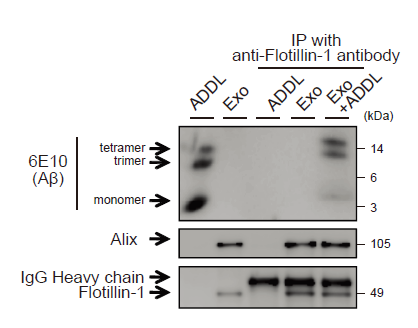
**

**Supplemental Figure 1. *In vitro* Pull-down of exosome-bound ADDLs.**  ADDLs or exosomes or the mixture of both were immunoprecipitated with an anti-Flotillin-1 antibody after *in vitro* incubation. To ascertain their direct binding, we immunoisolated exosomes using an antibody against Flotillin-1 after the incubation of exosomes with ADDLs and found that Aβ co-precipitated with those isolated exosomes. The detection of Alix in the “pulled-down” fraction indicated that we have immunoprecipitated intact exosomes but not exosome fragments or free Flotillin-1.
